# Estimation of overdiagnosis using short-term trends and lead time estimates uncontaminated by overdiagnosed cases: Results from the Norwegian Breast Screening Programme

**DOI:** 10.1177/0969141315623980

**Published:** 2016-03-02

**Authors:** Dimitrios Michalopoulos, Stephen W Duffy

**Affiliations:** Centre for Cancer Prevention, Wolfson Institute of Preventive Medicine, Queen Mary, University of London, London, UK

**Keywords:** Breast cancer screening, overdiagnosis, breast cancer incidence

## Abstract

**Background:**

Estimating overdiagnosis in cancer screening is complicated. Using observational data, estimation of the expected incidence in the screening period and taking account of lead time are two major problems.

**Methods:**

Using data from the Cancer Registry of Norway and the Norwegian Breast Cancer Screening Programme, we estimated incidence trends, using age-specific trends by year in the pre-screening period (1985–95). We also estimated sojourn time and sensitivity using interval cancers only. Thus, lead time estimates were uncontaminated by overdiagnosed cases. Finally, we derived estimates of overdiagnosis separately for all cancers, and for invasive cancers only, correcting for lead time, using two different methods.

**Results:**

Our results indicate that overdiagnosis of all cancers, invasive and in situ, constituted 15–17% of all screen-detected cancers in 1996–2009. For invasive cancers only, the corresponding figures were -2 to 7% in the same period, suggesting that a substantial proportion of the overdiagnosis in the Norwegian Programme was due to ductal carcinoma in situ.

**Conclusion:**

Using short-term trends, instead of long, prior to screening was more effective in predicting incidence in the screening epoch. In addition, sojourn time estimation using symptomatic cancers only avoids over-correction for lead time and consequently underestimation of overdiagnosis. Longer follow-up will provide more precise estimates of overdiagnosis.

## Introduction

Overdiagnosis in the context of cancer screening is the diagnosis, as a result of screening, of cancer which would not have been diagnosed in the lifetime of the host if screening had not taken place.^1^ An ideal estimate of overdiagnosis could be derived from a randomized trial of screening in which the control group was never screened and both groups were followed up to 100% expiry. In the absence of trial data, a way to estimate overdiagnosis is from trends in observational data on national or regional incidence of breast cancer, in conjunction with the time of introduction of screening.^2–5^ Researchers often estimate trends in incidence prior to screening and project these to predict incidence during the screening period. An excess between the observed and the predicted incidence may be partly attributable to overdiagnosis. However, some of the excess will also be due to lead time, the diagnosis as a result of screening of cancers which would otherwise have been diagnosed symptomatically some years later.

There are two major problems to be overcome in estimation of overdiagnosis from observational data: estimation of the incidence to be expected in the absence of screening and taking account of lead time.^6^ To be effective, screening has to detect substantial numbers of cancers a number of years earlier than they would have been diagnosed due to symptoms, so there is inevitably an observed excess incidence in a screened population. To separate the excess due to earlier diagnosis from that due to overdiagnosis requires either long follow-up or estimation of the likely lead time of the screen-detected tumours. It is desirable that the lead time estimates should not be from screen-detected cancers, as these will include overdiagnosed tumours.^7^ The lead time is a function of the mean sojourn time, the duration of the preclinical screen-detectable period.

In this paper, we used data from the Cancer Registry of Norway and the Norwegian Breast Cancer Screening Programme (NBCSP) to estimate overdiagnosis. We compared observed and expected cancers in the screening programme adjusted for trends in incidence and lead time. The estimates of lead time were derived entirely from interval cancers, and therefore do not include any overdiagnosed cases. Thus, there was no over-correction due to overdiagnosed cancers being used in the lead time estimates.

## Data and methods

The NBCSP started in November 1995, offering biennial two-view mammography to women aged 50–69, a population varying around 500,000 women. The programme began in four of the 19 counties in Norway and achieved nationwide coverage of invitation in 2005. Women receive a personal invitation by post every two years, regardless of their cancer history.^8^ Mammography is carried out in specialist breast centres, and mammograms are double read. In 1995, only 956 screens took place and there were only three screen-detected cancers. We therefore included 1995 in our nominal pre-screening period. By the end of 2000, 39% of the eligible population had been screened at least once. By the end of 2005, the figure was 88%. Attendance at screening varies around 76%.^9^

Data were supplied by the Cancer Registry of Norway under strict confidentiality and non-disclosure conditions. We obtained data on breast cancers, invasive and in situ from the Cancer Registry of Norway, including age at and date of diagnosis, from 1953 to 2009 (ductal carcinoma in situ (DCIS) was only registered from 1993 onwards). The NBCSP provided data on detection mode (outside of the screening cohort, screen detected, interval cancer, non-attender, not invited due to upper age limit and not invited as opted out). From the NBCSP, we had data on all screening invitations and attendances from November 1995 to December 2009. We also had tabular data on the resident female population in Norway by age and calendar year, as estimated in January every year. Age was calculated by subtracting the date of birth from the relevant calendar time.

We first estimated log-linear trends in incidence rates, per individual calendar year within each five-year age group from 50–54 to 80–84, using data from years 1985–95, by fitting a Poisson regression model in each age group of the form

ln(c)=a+bx+ln(P)

where c is the number of cases in a given year, x is the year and P is the person-years at risk within that year. Thus, b is the trend in increasing log incidence with time.

Duffy et al. noted that long-term pre-screening trends did not give good prediction of the incidence in the screening period.^10^ We therefore followed the approach of Moller et al.^11^ and used only the 11 years pre-screening, 1985–95. We fitted Poisson regression models to these within each age group as noted above. The numbers of cases and person-years by year and age group used to estimate the trends are shown in Table 1. We projected the trends b in the model above to give predicted incidence rates by age group in the periods 1996–2000, 2001–5 and 2006–9.

**Table 1.** Cases, person-years and incidence rate per 100,000 (in that order) by five-year age group and one-year calendar period. Incidence rates are shown in bold.

	1985	1986	1987	1988	1989	1990
**30–34**	22	25	23	25	34	25
	149,215	151,942	153,049	154,082	154,921	154,716
	**14.74**	**16.45**	**15.03**	**16.23**	**21.95**	**16.16**
**35–39**	80	67	51	68	68	68
	153,066	148,981	147,437	147,728	148,740	149,955
	**52.27**	**44.97**	**34.59**	**46.03**	**45.72**	**45.35**
**40–44**	132	133	116	127	128	145
	127,044	138,884	146,841	151,804	153,098	152,902
	**103.90**	**95.76**	**79.00**	**83.66**	**83.61**	**94.83**
**45–49**	131	136	146	136	149	172
	101,342	103,055	106,780	111,565	118,529	126,359
	**129.27**	**131.97**	**136.73**	**121.90**	**125.71**	**136.12**
**50–54**	131	120	113	139	109	131
	95,175	93,653	93,144	94,980	97,487	100,154
	**137.64**	**128.13**	**121.32**	**146.35**	**111.81**	**130.80**
**55–59**	133	180	134	140	122	127
	104,604	102,171	100,738	97,841	95,685	93,362
	**127.15**	**176.18**	**133.02**	**143.09**	**127.50**	**136.03**
**60–64**	198	213	180	181	183	156
	117,192	113,995	110,102	106,550	103,279	101,408
	**168.95**	**186.85**	**163.48**	**169.87**	**177.19**	**153.83**
**65–69**	222	235	238	231	224	214
	114,546	116,282	116,441	116,331	116,690	111,885
	**193.81**	**202.09**	**204.40**	**198.57**	**191.96**	**191.27**
**70–74**	220	208	225	230	244	235
	100,411	100,954	102,078	103,119	102,616	106,104
	**219.10**	**206.03**	**220.42**	**223.04**	**237.78**	**221.48**
**75–79**	211	189	209	221	223	224
	82,082	83,347	84,959	86,146	86,646	87,415
	**257.06**	**226.76**	**246.00**	**256.54**	**257.37**	**256.25**
**80–84**	142	148	135	143	158	186
	58,208	59,200	60,337	61,226	62,634	63,965
	**243.95**	**250.00**	**223.74**	**233.56**	**252.26**	**290.78**
**85–89**	89	76	76	87	78	84
	31,832	32,943	33,904	35,245	36,183	36,852
	**279.59**	**230.70**	**224.16**	**246.84**	**215.57**	**227.94**
	1991	1992	1993	1994	1995	
**30–34**	35	27	38	28	39	
	154,168	154,420	155,650	157,715	160,391	
	**22.61**	**17.48**	**24.41**	**17.75**	**24.32**	
**35–39**	76	81	99	78	82	
	152,754	153,792	154,698	155,663	155,861	
	**49.75**	**52.67**	**64.00**	**50.11**	**52.61**	
**40–44**	147	129	157	169	154	
	148,637	147,194	147,492	148,716	150,395	
	**98.90**	**87.64**	**106.45**	**113.64**	**102.40**	
**45–49**	195	219	239	228	282	
	138,021	145,869	150,794	152,443	152,512	
	**141.28**	**150.13**	**158.49**	**149.56**	**184.90**	
	1991	1992	1993	1994	1995	
**50–54**	153	137	182	197	210	
	101,862	105,526	110,351	117,402	125,303	
	**150.20**	**129.83**	**164.93**	**167.80**	**167.59**	
**55–59**	153	150	148	207	169	
	91,907	91,528	93,376	95,942	98,745	
	**166.47**	**163.88**	**158.50**	**215.76**	**171.15**	
**60–64**	201	183	194	162	214	
	99,125	97,798	95,085	93,090	90,973	
	**202.77**	**187.12**	**204.03**	**174.03**	**235.23**	
**65–69**	215	196	188	196	208	
	108,778	105,075	101,720	98,667	97,084	
	**197.65**	**186.53**	**184.82**	**198.65**	**214.25**	
**70–74**	241	256	246	252	250	
	107,640	107,887	108,019	108,429	104,016	
	**221.83**	**237.29**	**226.27**	**232.41**	**240.35**	
**75–79**	221	225	226	236	226	
	87,911	89,124	90,185	89,930	93,373	
	**251.39**	**252.46**	**250.60**	**262.43**	**242.04**	
**80–84**	150	192	150	161	181	
	65,110	66,262	67,449	67,885	68,776	
	**230.38**	**289.76**	**222.39**	**237.17**	**263.17**	
**85–89**	106	90	88	94	105	
	37,448	38,465	39,245	40,299	41,330	
	**283.06**	**233.98**	**224.23**	**233.26**	**254.05**	

Day^12^ derives the expected incidence of symptomatic cancer in the year following a screen as

$$E=I\int_{0}^{1}F(t)\text{d}t+I(1-S)\int_{0}^{1}(1-F(t))\text{d}t$$

where I is the expected incidence in the absence of screening, S is the screening sensitivity and F is the distribution function of the sojourn time. The first component is the incidence of cancers which have entered the preclinical screen detectable period after the screen and progressed to symptomatic disease by one year from the screen. The second component is the incidence of cancers which were already in the preclinical screen-detectable phase at the time of the screen but which were missed by the screen (hence the 1-S in the equation), then subsequently progressed to symptomatic disease within one year of the screen. The above simplifies to

$$E=I(1-S)+\mathrm{IS}\int_{0}^{1}F(t)\text{d}t$$

If the sojourn time is distributed as exponential with mean 1/λ, which fits breast cancer data reasonably well,^13^ this becomes

$$E=I(1-S)+\mathrm{IS}\{\frac{\lambda +e^{-\lambda }-1}{\lambda }\}$$

This differs slightly from the more complex formulae in Duffy et al.^14^ as the latter apply to a general time t, not necessarily one year, and assume that tumours can remain in the preclinical detectable phase for several rounds of screening and be missed at each successive round. If we have c symptomatic cancers occurring in the year after a screen of N subjects, the log-likelihood, assuming a Poisson distribution, is

ln(L)=cln(EN)-EN

We maximized this log-likelihood with respect to S and λ with three realizations of c and N – the numbers of interval cancers within a year of screening and numbers of women screened in each of the three periods 1996–2000, 2001–5 and 2006–9. We estimated I as the expected incidence projecting the pre-screening trend in incidence from the 11 years prior to screening as noted above. It was not possible to derive closed-form maximum likelihood estimates, so we derived them by calculating all possible values of the log-likelihood over a grid of values of λ and S.^13^ From the log-likelihood, we derived profile likelihood confidence intervals on S and λ.^15^

The estimation of mean sojourn time and therefore lead time was entirely from data on symptomatic cancers, and therefore did not include overdiagnosed tumours. The estimation was carried out separately for the five-year age groups 50–54, 55–59, 60–64 and 65–69.

We estimated overdiagnosis by two methods.

### Method 1

First, we calculated the excess numbers of cancers diagnosed in ages 50–69 in 1996–2009, compared with the expected numbers from the trends in the pre-screening periods, minus any deficit in ages 70–84 compared with expected numbers from the pre-screening trends. We then used the sojourn time estimates to further subtract from the excess any screen-detected cancers expected to be symptomatically diagnosed after the period of observation (i.e. after 2009). For screen-detected cancers diagnosed in 1996–2000, the average proportion which would be expected to be symptomatically diagnosed after 2009 would be

$$e^{-11.5\lambda }$$

because the average time to the end of 2009 is 11.5 years. Similarly, the proportions of screen-detected cancers diagnosed in 2001–5 and 2006–9 would be

$$e^{-6.5\lambda }\text{and}e^{-2\lambda }$$

### Method 2

The second method of estimation used the fact that the expected number of screen-detected cancers at a prevalent screen is

$$\frac{N_{p}\mathrm{IS}}{\lambda }$$

and the expected number at an incident screen is

$$N_{i}\{\frac{\mathrm{SI}(1-e^{-t\lambda })}{\lambda }+\frac{\mathrm{SI}(1-e^{-t\lambda })}{\lambda }[\frac{(1-S)e^{-t\lambda }}{1-(1-S)e^{-t\lambda }}]\}$$

where N_p_ is the number of prevalent screens, N_i_ the number of incident screens and t the interscreening interval, in this case two years. The last formula simplifies to

$$\frac{N_{i}\mathrm{SI}(1-e^{-2\lambda })}{\lambda }\{\frac{1}{1-(1-S)e^{-2\lambda }}\}$$

The formula above differs from the round-specific formulae in Duffy et al.^14^ for two reasons. First, because the sensitivity and sojourn time estimates are explicitly estimated from non-overdiagnosed cancers, the formula does not include a term for overdiagnosed cancers. Second, we made the simplifying assumption that a common incidence screen detection rate would apply, based on the steady-state estimate of the programme sensitivity, that is the proportion of incident cancers expected to be screen detected.^16^ The mathematical details are given in the Appendix, available online. If we then subtract the expected numbers at prevalent and incident screens from those observed, the remainder is an estimate of the overdiagnosed cases.

These methods are best seen by illustration, as in the results below. We present, in order

1. Results for all cancers, invasive and in situ, method 1.
2. Results for all cancers, invasive and in situ, method 2.
3. Results for invasive cancers only, method 1.
4. Results for invasive cancers only, method 2.

## Results

### All cancers

#### Method 1

We first estimate overdiagnosis from all cancers, invasive and DCIS. Table 2 shows the observed numbers of breast cancers by age and period from 1996 to 2009, and expected numbers calculated by extrapolation of the annual age-specific log-linear trends in 1985–95, for ages 50–84. There were substantial excesses of cancers in the age groups 50–69 and smaller deficits at ages 70–84. The excesses at ages 50–54, 55–59, 60–64 and 65–69 were, respectively, 1108, 1280, 1197 and 1728. The deficits at ages 70–74, 75–79 and 80–84 were 287, 99 and 24. Adding the deficits observed in women aged 70–74 in 2001–2009 and 75–79 in 2006–2009 gives a deficit of 383 in cohorts which were eligible for screening after the start of the programme. Although not all of the women in these cohorts will have actually been exposed to screening, it is worth noting that this constitutes 93% (383/410) of the deficit above the age range for screening, suggesting that this deficit is indeed chiefly due to cancers detected earlier by screening.

**Table 2.** Observed and expected breast cancers, invasive and in situ, by age and period, with person-years at risk-expected cases from 11-year time trends.

Age	Quantity	Period			
		1996–2000	2001–2005	2006–2009	Total
50–54	Observed cancers	1806	1970	1639	5415
	Expected cancers	1314	1529	1464	4307
	Person-years	733,226	736,185	617,197	2,086,608
55–59	Observed cancers	1576	2408	1628	5612
	Expected cancers	1104	1680	1548	4332
	Person-years	552,441	721,321	579,386	1,853,148
60–64	Observed cancers	1316	1906	1807	5029
	Expected cancers	1009	1317	1506	3832
	Person-years	460,508	538,061	558,423	1,556,992
65–69	Observed cancers	1355	1564	1372	4291
	Expected cancers	900	871	792	2563
	Person-years	457,065	442,206	402,346	1,301,617
70–74	Observed cancers	1195	946	730	2871
	Expected cancers	1168	1102	888	3158
	Person-years	477,081	428,140	329,812	1,235,033
75–79	Observed cancers	1200	1082	747	3029
	Expected cancers	1211	1101	816	3128
	Person-years	474,740	426,331	312,682	1,213,753
80–84	Observed cancers	902	1030	766	2698
	Expected cancers	932	1019	771	2722
	Person-years	360,633	386,082	286,097	1,032,812
Total	Observed cancers	9350	10,906	8689	28,945
	Expected cancers	7638	8619	7785	24,042
	Person-years	3,515,694	3,678,326	3,085,943	10,279,963

Table 3 shows the interval cancers arising within one year of a screen, number of screens prior to the interval cancer incidence and expected incidence from the extrapolated 1985–95 trends, by age group and period, with the maximum likelihood estimates of λ and S derived from these values.

**Table 3.** Numbers of interval cancers within one year of screening, numbers of screens and expected annual incidence from 11-year pre-screening trends, by age and period, with the maximum likelihood estimates of λ and S from the interval cancer data.

Age	Period	One-year interval cancers	Number of screens	One-year interval cancer rate	Expected annual incidence	Proportionate interval cancer rate	Estimate of λ (95% CI)	Estimate of S (95% CI)
50–54	1996–2000	46	106,661	0.00043	0.001792	0.24	0.33 (0.26–0.41)	0.88 (0.85–0.91)
	2001–2005	124	225,343	0.00055	0.002077	0.26		
	2006–2009	118	206,848	0.00057	0.002372	0.24		
55–59	1996–2000	46	85,072	0.00054	0.001998	0.27	0.23 (0.16–0.31)	0.84 (0.81–0.87)
	2001–2005	149	237,884	0.00063	0.002329	0.27		
	2006–2009	126	210,214	0.0006	0.002672	0.22		
60–64	1996–2000	41	71,624	0.00057	0.002191	0.26	0.43 (0.37–0.51)	0.99 (0.96–1.00)
	2001–2005	85	178,912	0.00048	0.002448	0.20		
	2006–2009	98	204,408	0.00048	0.002697	0.18		
65–69	1996–2000	27	65,897	0.00041	0.001969	0.21	0.11 (0.01–0.24)	0.75 (0.71–0.79)
	2001–2005	83	132,407	0.00063	0.001970	0.32		
	2006–2009	78	131,161	0.00059	0.001968	0.30		

Table 4 shows the numbers of screen-detected cancers by age group and period, and the proportions and numbers of these expected not to arise symptomatically until after the end of 2009. For example, of the 480 screen-detected cancers diagnosed at ages 50–54 in 1996–2000, the expected percentage to arise symptomatically after the end of 2009 is 100 × e^−(11−5×0.33)^ = 2.25%. The expected number which would not have been diagnosed until after the period of observation is therefore 480 × 0.0225 = 11 cancers.

**Table 4.** Total screen-detected cancers, percentages and numbers of screen-detected cancers expected not to have been diagnosed symptomatically until after 2009.

Age	Period	Screen- detected cancers	Percentage expected symptomatic after 2009	Number expected symptomatic after 2009
50–54	1996–2000	480	2.25	11
	2001–2005	969	11.71	113
	2006–2009	901	51.69	466
55–59	1996–2000	434	7.10	31
	2001–2005	1341	22.42	301
	2006–2009	955	63.13	603
60–64	1996–2000	447	0.71	3
	2001–2005	1075	6.11	66
	2006–2009	1172	42.32	496
65–69	1996–2000	472	28.22	133
	2001–2005	863	48.92	422
	2006–2009	905	80.25	726
Total		10014	33.66	3371

The total excess cancers diagnosed at ages 50–69 over that expected from pre-screening trends was 5313 (1108 + 1280 + 1197 + 1728). Subtracting the 410 deficit observed at ages 70–84 and the 3371 screen-detected cancers expected to arise symptomatically after 2009 gives a lead time adjusted excess of 1532 cancers. This may be regarded as an estimate of the number of overdiagnosed cancers, but there are uncertainties and qualifications to this (see ‘Discussion’ section). This represents 5% of the 28,945 cancers diagnosed in women aged 50–84 between 1996 and 2009; 8% of the 20,347 cancers diagnosed in women in the screening age range, 50–69, in the same period; and 15% of the 10,014 screen-detected cancers. A woman attending all 10 screens from age 50 to 69 would have roughly a 5.4% chance of a screen-detected cancer, given the average detection rate of 5.4 per thousand. Thus, she would have a risk of an overdiagnosed tumour of 8 per thousand (0.15 × .054).

#### Method 2

To estimate overdiagnosis by our second method, we need the numbers of prevalent and incident screens by age group and period, in our screening period 1996–2009. Table 5 shows the numbers of prevalent and incident screens, and the expected yields of cancers from these, by age and period. The expected numbers of cancers are calculated as described above. For example, for age group 50–54 with λ estimated as 0.33, S as 0.88 and underlying annual incidence as 0.001792, the expected number of prevalent screen cancers is

$$\frac{71786\times 0.001792\times 0.88}{0.33}=343$$

**Table 5.** Prevalent screens, incident screens and expected numbers of screen-detected cancers by age and period.

Age group	Period	Prevalent screens	Expected cancers	Incident screens	Expected cancers
50–54	1996–2000	71,786	343	34,875	86
	2001–5	122,183	677	103,160	294
	2006–9	82,248	520	124,600	406
55–59	1996–2000	40,443	295	44,629	134
	2001–5	65,535	557	172,349	601
	2006–9	7184	70	203,030	813
60–64	1996–2000	34,571	174	37,053	108
	2001–5	47,686	269	131,226	428
	2006–9	4735	29	199,673	718
65–69	1996–2000	32,002	430	33,895	112
	2001–5	35,225	473	97,182	322
	2006–9	2615	35	128,546	426
Total		546,213	3872	1,310,218	4448

The expected number of incident screen cancers is

$$\frac{34875\times 0.88\times 0.001792\times (1-e^{-2\times 0.33})}{0.33}\;\times \{\frac{1}{1-(1-0.88)e^{-2\times 0.33}}\}=86$$

The total number of screen-detected cancers expected was 3872 + 4448 = 8320. Subtracting this from the 10,014 observed screen-detected cancers gives 1694 cancers estimated to be overdiagnosed, although again there are uncertainties and qualifications to this (see ‘Discussion’ section). This would represent 6% of cancers diagnosed at ages 50–84, 8% of cancers diagnosed at ages 50–69 and 17% of screen-detected cancers. This would translate to an absolute risk of nine per thousand in a woman attending all scheduled screens in the programme.

### Invasive cancers only

#### Method 1

We then estimate overdiagnosis from invasive cancers only. Table 6 shows the observed numbers of invasive breast cancers by five-year age and period groups from 1996 to 2009; expected numbers calculated by projecting the annual age-specific log-linear trends in 1985–95 and person-years for ages 50–84. As with the total cancers, invasive and DCIS, significant excess numbers of invasive cancers were observed in the screening age groups 50–69, and smaller deficits above the screening age groups 70–84. The excesses at ages 50–54, 55–59, 60–64 and 65–69 were, respectively, 935, 1020, 885 and 1360. The deficits at ages 70–74, 75–79 and 80–84 were 288, 124 and 23. Table 7 shows the invasive interval cancers diagnosed within one year of a screen, numbers of screens and expected annual incidence from pre-screening trends, by age and period, with the maximum likelihood estimates of λ and S derived from the interval cancer data.

**Table 6.** Observed and expected breast cancers, invasive only, by age and period, with person-years at risk-expected cases from 11-year time trends.

Age	Quantity	Period			
		1996–2000	2001–2005	2006–2009	Total
50–54	Observed cancers	1598	1719	1386	4703
	Expected cancers	1199	1337	1232	3768
	Person-years	733,226	736,185	617,197	2,086,608
55–59	Observed cancers	1412	2099	1434	4945
	Expected cancers	1034	1525	1366	3925
	Person-years	552,441	721,321	579,386	1,853,148
60–64	Observed cancers	1202	1684	1581	4467
	Expected cancers	965	1233	1384	3582
	Person-years	460,508	538,061	558,423	1,556,992
65–69	Observed cancers	1224	1397	1197	3818
	Expected cancers	874	834	750	2458
	Person-years	457,065	442,206	402,346	1,301,617
70–74	Observed cancers	1124	890	684	2698
	Expected cancers	1123	1039	824	2986
	Person-years	477,081	428,140	329,812	1,235,033
75–79	Observed cancers	1159	1040	694	2893
	Expected cancers	1181	1060	776	3017
	Person-years	474,740	426,331	312,682	1,213,753
80–84	Observed cancers	881	1005	749	2635
	Expected cancers	916	995	747	2658
	Person-years	360,633	386,082	286,097	1,032,812
Total	Observed cancers	8600	9834	7725	26,159
	Expected cancers	7292	8023	7079	22,394
	Person-years	3,515,694	3,678,326	3,085,943	10,279,963

**Table 7.** Numbers of invasive interval cancers within one year of screening, numbers of screens and expected annual incidence from pre-screening trends, by age and period, with the maximum likelihood estimates of λ and S from the interval cancer data.

Age	Period	One-year interval cancers	Number of screens	One-year interval cancer rate	Expected annual incidence	Proportionate interval cancer rate	Estimate of λ	Estimate of S
50–54	1996–2000	44	106,661	0.00041	0.001635	0.25	0.26 (0.18–0.35)	0.83 (0.80–0.86)
	2001–2005	114	225,343	0.00051	0.001816	0.28		
	2006–2009	110	206,848	0.00053	0.001996	0.27		
55–59	1996–2000	43	85,072	0.00051	0.001872	0.27	0.46 (0.38–0.55)	0.92 (0.89–0.95)
	2001–2005	141	237,884	0.00059	0.002114	0.28		
	2006–2009	120	210,214	0.00057	0.002358	0.24		
60–64	1996–2000	40	71,624	0.00056	0.002096	0.27	0.10 (0.04–0.17)	0.84 (0.82–0.86)
	2001–2005	83	178,912	0.00046	0.002292	0.20		
	2006–2009	91	204,408	0.00045	0.002478	0.18		
65–69	1996–2000	25	65,897	0.00038	0.001912	0.20	0.17 (0.06–0.30)	0.77 (0.73–0.81)
	2001–2005	80	132,407	0.0006	0.001886	0.32		
	2006–2009	76	131,161	0.00058	0.001864	0.31		

The numbers of invasive screen-detected cancers by five-year age and period groups and the percentages and numbers of invasive screen-detected cancers not to have been diagnosed symptomatically until after 2009 are shown in Table 8.

**Table 8.** Total invasive screen-detected cancers, percentages and numbers of invasive screen-detected cancers expected not to have been diagnosed symptomatically until after 2009.

Age	Period	Invasive screen-detected cancers	Percentage expected symptomatic after 2009	Number expected symptomatic after 2009
50–54	1996–2000	379	5.03	19
	2001–2005	797	18.45	147
	2006–2009	696	59.45	414
55–59	1996–2000	342	0.50	2
	2001–2005	1100	5.03	55
	2006–2009	796	39.85	317
60–64	1996–2000	384	31.66	122
	2001–2005	913	52.20	477
	2006–2009	979	81.87	802
65–69	1996–2000	394	14.16	56
	2001–2005	735	33.12	243
	2006–2009	754	71.18	537
Total		8269	38.58	3190

The total excess of invasive cancers diagnosed at ages 50–69 over that expected from pre-screening trends was 4200 (935 + 1020 + 885 + 1360). Subtracting the deficit of 435 cancers observed at ages 70–84 and the 3190 screen-detected cancers expected to be diagnosed symptomatically after 2009 gives a lead time adjusted excess of 575 cancers. This represents 2% of the 26,159 invasive cancers diagnosed in women aged 50–84 between 1996 and 2009; 3% of the 17,933 invasive cancers diagnosed in women in the screening age range, 50–69, in the same period; and 7% of the 8269 invasive screen-detected cancers. This would mean an absolute risk of three per thousand of an overdiagnosed invasive tumour in a woman attending all scheduled programme screens.

#### Method 2

To estimate overdiagnosis by our second method, we again use the numbers of prevalent and incident screens by age group and period, in our screening period 1996–2009. Table 9 shows the numbers of prevalent and incident screens, and the expected invasive cancers diagnosed from these, by age and period.

**Table 9.** Prevalent screens, incident screens and expected numbers of invasive screen-detected cancers by age and period.

Age group	Period	Prevalent screens	Expected cancers	Incident screens	Expected cancers
50–54	1996–2000	71,786	375	34,875	82
	2001–5	122,183	708	103,160	270
	2006–9	82,248	524	124,600	358
55–59	1996–2000	40,443	151	44,629	104
	2001–5	65,535	277	172,349	453
	2006–9	7184	34	203,030	595
60–64	1996–2000	34,571	609	37,053	136
	2001–5	47,686	918	131,226	527
	2006–9	4735	99	199,673	867
65–69	1996–2000	32,002	277	33,895	101
	2001–5	35,225	301	97,182	286
	2006–9	2615	22	128,546	374
Total		546,213	4295	1,310,218	4153

The total number of invasive screen-detected cancers expected was 4295 + 4153 = 8448. Subtracting this from the 8269 observed invasive screen-detected cancers gives a deficit of 179 cancers. This suggests that there is no overdiagnosis of invasive cancers only. Because the first method gave an estimate of 575 cancers overdiagnosed (7% of screen detected), the true value is likely to lie between the two.

## Discussion

Overdiagnosis in cancer screening is notoriously difficult to estimate. As is common practice in the physical sciences, when a quantity is difficult to measure, we measure it more than once and by different methods. Both methods took account of lead time effects and (relatively) short-term pre-screening incidence trends. Our first method calculated the total observed cancers in the screening period and age range, and subtracted from these the total expected from pre-screening trends, the deficit observed above the screening age range and the number of screen-detected cancers which would have been expected to arise symptomatically only after the period of observation, but the diagnosis of which was brought forward to our period of observation by lead time. The result of this subtraction was our first estimate of overdiagnosis. The second method took advantage of the fact that only screen-detected cancers can be overdiagnosed. It calculated expected numbers of screen-detected cancers at prevalent and incident screens, based on underlying incidence projected from pre-screening trends, estimated screening sensitivity and sojourn time. The excess of total observed screen-detected cancers over total expected gave a second estimate of overdiagnosis. The estimates from Methods 1 and 2 are not independent, being based on the same estimates of sensitivity and mean sojourn time. Thus, it might be expected that they would be of similar magnitude. One might argue that Method 2 is to be preferred as being the more direct. However, when a quantity can never be measured perfectly, it is desirable to measure it more than once, using different methods.

The use of projected incidence rates from the pre-screening period to estimate the underlying incidence has a crucial rationale in two areas. First, it means that there is an estimate of excess incidence compared with an independent estimate of the expected incidence in the absence of screening. Second, it affords estimation of sojourn time using cancers which were not screen detected (and therefore by definition not overdiagnosed), in that in addition to using only interval cancers from the screening period, the underlying incidence estimate was derived from pre-screening data. Thus, we avoided over-correction for lead time (and consequent underestimation of overdiagnosis) arising from use of lead time estimates which include overdiagnosed cancers.^7^

These results suggest that overdiagnosis of all cancers, invasive and in situ, constituted 5–6% of cancers diagnosed in women aged 50–84 in 1996–2009 and 15–17% of screen-detected cancers in the same period. For invasive cancers alone, the corresponding figures were 0–2% of invasive cancers diagnosed at age 50–84 and 0–7% (indeed one estimate was −2%) of screen-detected cancers. This suggests that most of the overdiagnosis in the Norwegian programme was due to DCIS. There are a number of qualifications to these estimates. First, while the estimates of λ tend generally to be smaller (implying longer lead times) for older subjects, they do not fall monotonically with age. Similarly, we did not observe a clear trend of increasing sensitivity with age. The restriction to interval cancer rates as the data resource for estimation probably adds an element of uncertainty. Second, we had to make the assumption that sojourn time in interval cancers is the same as sojourn time in a general unscreened population. Due to the converse of length bias, interval cancers may have a shorter sojourn time than the general tumour population. If this is the case, however, our estimates will be conservative, so they will not lead to underestimation of overdiagnosis. Third, it would be useful to have a longer period of screening exposure to study, which would give better estimates of the deficit, if any, after screening stops.

Some unusual observations arise in the data. First, the number of cancers at ages 55–59, especially but not exclusively in 2001–2005, is particularly high (Tables 2, 4, 6 and 8). This is largely due to the considerable amount of screening activity, especially incident screening, in this age group (Table 5). Second, under our second method, there was a higher expected number of screen-detected invasive cancers than the expected total. This was due to a particularly high estimated number of prevalent screen cancers at ages 60–64, which was in turn due to the very low estimate of λ for this group (Tables 7 and 9). The upper confidence interval on λ for this group would reduce the expected screen-detected invasive cancers to well below the expected number for total cancers invasive and in situ. This suggests that uncertainty in estimation of λ, and sensitivity of expected numbers to the estimate, is a limitation of this study.

However, it should be noted that whatever method is used to estimate overdiagnosis, the longer the period of observation, the better.^17^ Given that the definition of overdiagnosis pertains to the lifetime of the patient, long follow-up is clearly desirable. In our data, we have relatively little person-time in women exposed to screening but who are now above the screening age range. A further five years of observation would yield considerable data on women in whom screening has stopped, an invaluable data source for estimation of overdiagnosis. A target for the future is to investigate whether the post-screening deficit occurs earlier for the four counties which started screening earlier than in the rest of Norway.^8^

Excess incidence tended to be highest in the oldest screening age group, 65–69. This is consistent with overdiagnosis being greater at older ages, due to shorter future life expectancy and longer lead times. Interestingly, there was not a strong difference in proportions of in situ tumours by age (Tables 2 and 6). In 1996–2000, 12% of tumours in the 50–54 group were in situ compared with 10% at ages 65–69. The corresponding estimates for 2001–2005 were 13% versus 11%, and for 2006–2009 15% versus 13%. This suggests that there is a greater proportional contribution of invasive cancers to the higher overdiagnosis rates at older ages. We also used data on the use of hormone replacement therapy to predict the incidence rates in the screening period, but we obtained similar results. Our analysis was restricted to women of screening age (50–69). Whilst we acknowledge that some screen-detected cancers could occur outside of this age range, in our dataset, only 152 (1.5%) of the screen-detected cancers were diagnosed below age 50, and only 174 (1.7%) at ages 70 or more, thus with so few screen-detected cancers occurring outside of 50–69, we believe that only including women of screening age 50–69 is most appropriate. Our second method of estimation indicated that overdiagnosis was mainly a phenomenon of incident rather than prevalent screens, which is unusual.^1,2^ There were in total 546,213 prevalent screens, resulting in 2860 (5.2 per thousand) invasive cancers and 625 (1.1 per thousand) in situ, a total of 3485 cancers. There were 1,310,218 incident screens, with 5409 (4.1 per thousand) invasive cases detected and 1120 (0.9 per thousand) in situ, a total of 6529. The total expected prevalent cases was 3485, exceeding the observed numbers, whereas the total expected incident cases was 4448, suggesting a considerable excess of observed incident cancers. This may indicate a low sensitivity at the start of the programme, improving with time, as has been observed elsewhere.^18^ Also, the absolute number of DCIS cases diagnosed at incident screens was approximately double the number diagnosed at prevalent screening, and the percentage of screen-detected cancers which were DCIS was the same in incident and prevalent screens (details available from the authors). This contrasts with other programmes in which the proportion of DCIS is lower at incident screens.^19^

Our estimates of overdiagnosis are rather higher than those estimated by Njor et al. in the Danish breast screening programme.^20^ Also, inclusion of DCIS considerably increased our estimates, but did not significantly change estimates in the Danish programme. The first difference may be due to the longer follow-up since the start of screening in the Danish estimates. We suspect that with longer follow-up of the Norwegian programme, there will be greater opportunity to observe post-screening deficits, and more modest estimates of overdiagnosis will emerge. In considering the greater influence of DCIS in the Norwegian programme, it is worth noting that in the screening period in Norway, 9.6% of cancers were DCIS, whereas in Denmark the figures were 5.4% in Copenhagen and 5.8% in Funen.^20^ There may have been more aggressive workup of calcifications leading to greater diagnosis of DCIS in the Norwegian programme.

Overall our results indicated 1532–1692 cancers, invasive and in situ, overdiagnosed. This amounts to 15–17% of screen-detected cancers, and with the 1,856,431 screening episodes, one overdiagnosed cancer per 1100–1200 screening episodes, or one overdiagnosed cancer per 111–112 women attending all 10 scheduled screens between ages 50 and 69. The corresponding figures for invasive cancers only were -2 to 7% of screen-detected cancers, that is estimates ranging from no overdiagnosis at all to 575 overdiagnosed cancers, one per 3200 screening episodes. These figures require confirmation with longer follow-up in the screening period.
